# Using the articularis genu to test peri-articular muscle health during knee osteoarthritis

**DOI:** 10.1038/s41598-022-17046-w

**Published:** 2022-07-28

**Authors:** José A. Cruz Ayala, Mallory Crawford, Mary C. Gatterer, Maria Tovar, Jessica C. Rivera, Vinod Dasa, Luis Marrero

**Affiliations:** 1 School of Medicine, Louisiana State University Health Sciences Center, New Orleans, LA USA; 2 Department of Orthopaedic Surgery, Louisiana State University Health Sciences Center, New Orleans, LA USA; 3 Morphology and Imaging Core, Louisiana State University Health Sciences Center, New Orleans, LA USA

**Keywords:** Osteoarthritis, Skeletal muscle

## Abstract

Knee osteoarthritis (OA) involves peri-articular sarcopenia. The infrapatellar articularis genu (AG) links to the quadriceps femoris (QF) and can be sampled from discarded tissue during arthroplasty. We predict disuse-mediated changes in AG myofiber type ratio and atrophy similar to reports on the QF during OA. OA AGs (n = 40) were preserved and grouped by poor (≤ 85°; n = 11), fair (90°–110°; n = 19), and good (≥ 115°; n = 10) range of motion (ROM). Immunolabeling of slow and fast myosin heavy chains in AG sections allowed comparing distribution and cross-sectional area (CSA) of type-I (T1) and type-II (T2) myofibers between groups and associating to ROM. T1/T2 ratios in fair and poor ROM groups was consistent with those published in OA QF. Increasing mean ± SD T2 percentages from good (43.31 ± 11.76), to fair (50.96 ± 5.85), and poor (60.02 ± 8.29) ROM groups was significant between poor versus fair (*p* = 0.018) and good (*p* < 0.0001) in association with ROM deficits (r = − 0.729; *p* < 0.0001). T1 and T2 CSA decreased with worsening ROM, which associates with lower symptom scores (r = 0.3198; *p* = 0.0472). In-depth evaluation of the OA AG as a surrogate for the OA QF relative to serum and/or synovial fluid biomarkers of sarcopenia could refine diagnostics of peri-articular muscle health to guide individualized strength rehabilitation after surgery.

## Introduction

The quadriceps femoris (QF), a group of peri-articular muscles that extends the leg at the knee, is sensitive to joint disuse caused by osteoarthritis (OA)^1^. The QF is composed of the vastus lateralis, vastus intermedius, vastus medialis, and rectus femoris. In healthy individuals younger than 65 years of age, the myofiber composition throughout the QF is, on average, ~ 50% type-I (T1) slow-twitch myofibers, which use oxidative phosphorylation to fuel prolonged contraction. The remaining myofibers are categorized as type-II (T2) fast-twitch myofibers, which mostly drive ATP production via glycolysis to aid in sudden energy bursts, but fatigue easily^2^. The myofiber distribution and quality in each of the muscles of the QF can vary between individuals and is dependent on type and extent of exercise, age, and exposure to acute or chronic insults^3–6^. During OA, the disuse of the muscles affecting joint motion results in a decrease in the ratio of QF T1 to T2 concurrent with atrophy, one of the hallmarks of poor muscle health^7–10^.

The articularis genu *(*AG) is an intra-articular muscle of the knee originating on the anterior distal femur, deep to the vastus intermedius, and inserting onto the suprapatellar bursa^11^. The AG, sometimes referred to as the fifth muscle of the QF complex^9^, retracts the suprapatellar bursa during extension with similarly proportional myofiber distribution as the vastus lateralis and intermedius around healthy knees^11^. Consistent with studies on aging QFs^4,12^ the myofiber distribution of AGs from older individuals without OA disproportionately favors slow-twitch myofibers at a T1 to T2 ratio of ~ 2.3^9,11,13^.

Because the AG and each of the muscles of the QF co-activate as extensors of the knee, they all may be similarly sensitive to OA. This is particularly so because OA joint degeneration and synovial fibrosis can result in the loss of full knee extension, thereby decreasing the terminal joint angle at which the AG and QF are activated. Dysfunctional contraction of the AG has been measured by ultrasound in OA patients during isometric extension exercises to confirm that the OA AG atrophies in association with symptoms, pain, and deficits in range of motion (ROM)^9^. We hypothesize that the OA AG undergoes changes in myofiber distribution resembling published data on the OA QF. We also predict that changes in myofiber composition of the AG will associate with limitations in ROM, particularly flexion contracture (FxC). If true, our results would help establish a platform for more in-depth studies on the AG, following incidental removal during total knee arthroplasty (TKA), to determine the OA AG as a surrogate for OA QF, which could potentially help develop a diagnostic tool to test peri-articular muscle health at the time of surgery.

## Results

### Patient characteristics

Basic demographics, Kellgren-Lawrence  (KL) radiographic scores, ROM, and the knee injury and osteoarthritis outcome scores (KOOS) of 40 patients who underwent TKA, from whom AGs were harvested, are reported for the total sample with and without grouping by ROM (Table 1). The total patient sample was 60% female with median (range) age and BMI prior to surgery of 67 (47–81) years and 34 (22–42) kg/m^2^, respectively. Furthermore, 57.5% of the study population self-identified as white, 35% as black, and 7.5% as other race. There were no significant differences in mean BMI, age, KL scores, and KOOS subscale scores between ROM groups. ROM was measured pre-operatively by goniometer. Multiple comparisons of the mean ROM between groups were significant (*p* < 0.0001), with similar SDs (Table 1). We measured a moderately low but significant correlation between KOOS symptoms subscale scores and active ROM (r = 0.3198; *p* = 0.0472).

**Table 1:** Patient characteristics. Distribution of study participants by gender, race, and mean age, BMI, KL radiographic scores, KOOS and ROM for the total patient sample (n=40) or grouped by ROM status tested by one-way ANOVA or Kruskal-Wallis using Graphpad PRISM 9, in which *p* < 0.05 is significant.

			Range of motion						
			Good		Fair		Poor		*p*-value
	NT	Mean or *Median*	NG	Mean (SD) or *Median (IQR)*	NF	Mean (SD) or *Median (IQR)*	NP	Mean (SD) or *Median (IQR)*	
**Gender**									
Female	24	–	7	–	11	–	6	–	–
Male	16	–	3	–	8	–	5	–	–
**Race**									–
Black	14	–	4	–	6	–	4	–	–
White	23	–	6	–	11	–	6	–	–
Other	3	–	0	–	2	–	1	–	–
**Age (years)**									
	40	66.85	10	67.6 (9.09)	19	68.11 (8.94)	11	64.0 (9.37)	0.477
**BMI (kg/m2)**									
	40	33.19	10	33.7 (5.12)	19	32.4 (4.72)	11	34.2 (5.88)	0.631
**KL Score (0–4)**									
	40	3.93	10	3.90 (0.32)	19	3.95 (0.23)	11	3.91 (0.30)	0.883
**ROM**									
	40	99.88	10	125.50 (7.62)	19	99.74 (7.72)	11	76.82 (8.45)	< 0.0001
**KOOS**									
Symptoms	39	–	9	*43.00 (35.86–55.29)*	19	*43.00 (14.29–67.86)*	11	*32.14 (10.71–36.00)*	0.1823
Pain	39	–	9	*39.00 (27.50–51.61)*	19	*28.00 (16.67–47.00)*	11	*28.00 (22.22–47.00)*	0.4282
ADL	39	–	9	*38.00 (25.00–52.50)*	19	*31.00 (22.06–48.53)*	11	*31.00 (25.00–56.00)*	0.7268
QOL	39	–	9	*25.00 (12.38–31.00)*	19	*19.00 (6.00–37.50)*	11	*12.50 (0.00–37.50)*	0.4025

NT = total patient sample; NG = good ROM group; NF = fair ROM group; NP = poor ROM group.

### AG myofiber distribution and cross-sectional area (CSA) relative to ROM

**Figure 1:**
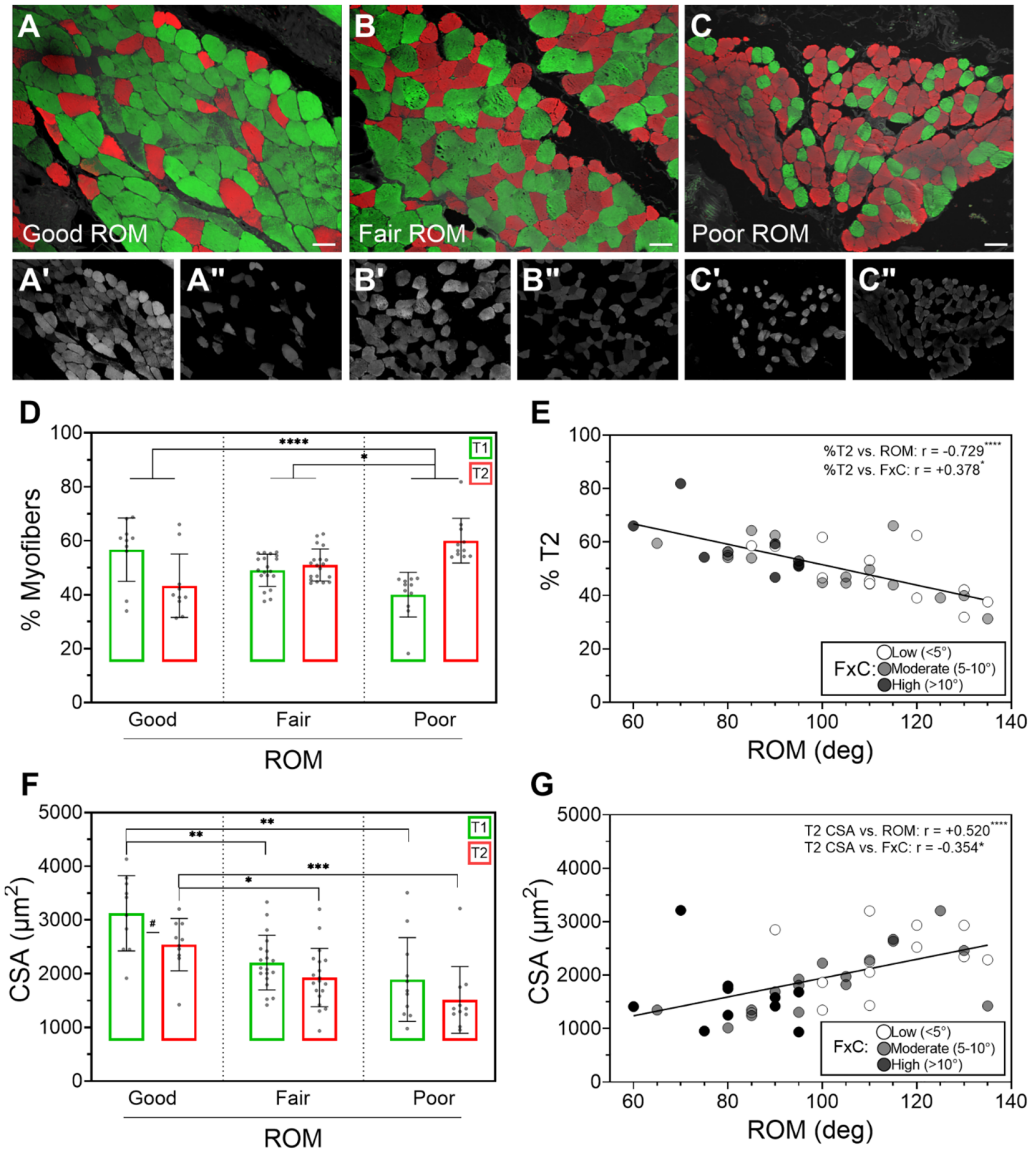
Myofiber distribution and CSA relative to ROM. Representative merged photomicrographs of co-labeled T1 (green), and T2 (red) myofibers in AG sections from (**A**) good, (**B**) fair, and (**C**) poor ROM patient groups were segmented by individual (**A**’–**C**’) T1, and (**A**”–**C**”) T2 grayscale channels for fiber count and CSA analyses using Slidebook 5 and Graphpad PRISM 9 software for morphometry and statistics, respectively. Bars = 50 µm. (**D**) Mean ± SD fiber counts between and within ROM groups. (**E**) Spearman’s ρ (r) was calculated between T2 (and correspondingly T1) fibers and total ROM. Data points were shaded according to severity of FxC, and r calculated between FxC and T2 content. (**F**) Mean CSAs were compared within and between ROM groups and (**G**) tested for associations with both total ROM and FxC. *p < 0.05 between groups and #*p* < 0.05 within groups; **p < 0.01; ***p < 0.001; and ****p < 0.0001.

The two major myofiber types were co-immunolabeled in AG sections grouped by ROM against fiber-specific myosin heavy chains (MHC) (Fig. 1A–C), which enabled automated quantification of sequentially captured slow MHC+ T1 (Fig. 1A’–C’) versus fast MHC+ T2 (Fig. 1A”–C”) myofibers. A ratio equivalent to 0.94 was calculated from the mean percentages of T1 (48.46%) over T2 (51.54%) content from values across the total patient sample. We did not measure significant differences between the mean percentages of myofiber types within the total patient sample (*p* = 0.180). However, when grouped by ROM status, the T1 over T2 ratio progressively decreased from good (1.31) to fair (0.96), and poor (0.67) ROM groups. A progressive (mean ± SD) increase in percentage of T2 fibers was measured from good (43.31 ± 11.76) to fair (50.96 ± 5.85), and poor (60.02 ± 8.29) ROM groups with correspondingly decreasing percentage of T1 (Fig. 1D). Differences in T2 percentages, and corresponding T1, were significant only when comparing the poor versus fair (*p* = 0.018) and poor versus good (*p* < 0.001) ROM groups. We calculated a moderately high, statistically significant, negative association (r = − 0.729; *p* < 0.0001) between ROM and percentage of T2 fibers in the total patient sample (Fig. 1E). Notably, a moderately low, significant association (r = 0.378; *p* = 0.0161) was measured between T2 content and severity of FxC (Fig. 1E).

Using CSA as a measure of AG myofiber atrophy (Fig. 1F), we recorded a generally smaller (mean ± SD) area (µm^2^) of T2 compared to (vs.) T1 fibers within each ROM group as follows: good (2541 ± 486.8 vs. 3123 ± 700.9); fair (1929 ± 542.5 vs. 2207 ± 508.0); and poor (1511 ± 620.1 vs. 1891 ± 779.9). T2 were significantly smaller than T1 only when compared within the good (*p* = 0.0447) ROM group. We measured a progressive decline in CSA values for both fiber types consistent with worsening ROM. T1 CSAs measured 39% and 29% significantly smaller in the poor (*p* = 0.0002) and fair (*p* = 0.0022) groups, respectively, when individually compared to the good ROM group. In a similar trend, T2 CSAs also measured significantly smaller in the poor (*p* = 0.0004) and fair (*p* = 0.0103) groups with 40% and 26% decrease, respectively, against the good ROM group. A statistically significant association was measured between CSA and ROM for both T1 (r = 0.585; *p* < 0.0001) and T2 (r = 0.520; *p* = 0.0006) in the total patient sample (Fig. 1G). In a similar trend, FxC severity negatively associated with T1 (r = − 0.292) and T2 (r = − 0.354) CSA but only the latter tested significant (*p* = 0.0252).

## Discussion

In this study, we employ metrics of structural changes in muscle phenotype to add to the limited knowledge on the sensitivity of the AG to OA relative to limitations in total arc of the knee. The AG can be easily accessed and sampled during TKA allowing our goal to continue banking and analyzing the OA AG relative to what has been reported on the OA QF. Establishing the OA AG as a surrogate to the OA QF can provide useful insight for potentially developing a diagnostic tool to assess the health status of the periarticular muscles around the time of TKA. Here, we begin our study by measuring the distribution and CSA of the major myofiber types in the AG of OA patients relative to similar studies on normal AG, and biopsies of QFs from healthy and OA patients^7,13^. While published data on aged, non-OA AGs suggest an increasing T1/T2 ratio, we identified a unique characteristic of muscle health decline in OA AGs consistent with decreasing T1/T2 ratios found in OA QFs. This phenomenon is magnified as OA patients exhibit worsening FxC. Also, in agreement with studies on disuse of the vastus lateralis and medialis, we measured preferential targeting of T2 fibers in OA AGs for atrophy^7,10^.

To support a predicted decline in AG quality during OA due to knee disuse, we started by testing myofiber distribution relative to ROM status. Our data show a mean 20% decrease in T1 myofibers in aged OA AGs against a reported 70% T1 content measured in older but non-OA AGs^13^. Compared to the myofiber type distribution in healthy vastus intermedius and lateralis^14,15^, and healthy AGs^13^, AGs from OA patients in our cohort with good ROM manifested higher T1 over T2 fiber ratios. Predominance of T1 fibers in the QF has been linked to aging^4,12^. A significantly decreasing ratio of T1 to T2 fibers corresponds to worsening ROM. In total, OA AGs from knees with good ROM have more similar T1/T2 ratios as non-OA, aged AGs. The reversal of this ratio manifests in knees with poor ROM, a harbinger of advancing OA pathology.

An increasing number of T2 fibers is generally associated with increases in muscle mass after strength training^16^. On the other hand, an elevated ratio of T2 over T1 fibers without increasing amount of total fibers, due to fiber type switching, has been implicated in muscular weakness associated with decreased activity. Evidence by Noehren et al. supports the trend in T1 to T2 switching in the vastus lateralis, including an increase in T2 fibers of the T2a/x hybrid subtype, which poses adverse functional implications^7^. In our sample, we measured increasing but atrophied T2 fibers relative to diminishing T1 content as ROM worsened. In addition to changes in myofiber type distribution, a targeted decrease in CSA of T2 fibers in the OA QF has been associated with joint disuse^17^. Our data show that the AG undergoes a similar trend, since we measured a significantly smaller CSA of T2 relative to T1 in the group of patients with good ROM, and an overall CSA decrease of both fiber types in proportion to higher ROM deficits. This indicates that although T2 fibers have been known to be the primary target of atrophy, T1 fibers are also vulnerable to disuse-related atrophy, similar to reports on the vastus medialis^10^.

As part of the family of leg extensors, understanding more specific differences within the gait cycle of each patient may offer clues to the structural changes that the AG and QF undergo due to OA-influenced disuse. Additionally, FxC of the knee requires more contractive force; a 15° flexion contracture leads to a 22% increase in the force needed for QF contraction^18^. During the typical gait cycle, the knee at terminal stance phase is fully extended, and QF contraction enacted at the muscles’ shortest working length. The morphology of the femoral-tibial articulation then results in the knee locking out, allowing for the QF to relax. Patients with OA also have FxC during their normal gait cycle when compared to adults without OA, resulting in loss of QF relaxation during terminal stance phase^19^. Clinically, this results in fatigue and exacerbates pain across the OA joint. Our data suggests that as T2 fibers in the AG increase, FxC also increases within our cohort. Changes in the fiber type composition of the AG that correlate with FxC may be indicative of myofiber type changes occurring in parallel to the QF, even though the AG has the smallest role around the QF extensor complex. As such, these histopathological findings in the AG could serve as a surrogate for QF fiber type aberrations as OA disease progression leads to functional loss of ROM. What is not known is whether the alterations in the muscle fiber type are a result of the knee joint loss of ROM or if the changes in the muscle contribute to decrements in terminal extension.

Our data suggest that the AG is sensitive to OA and portrays similar changes in myofiber-type distribution of disused OA QF. Changes in AG myofiber count also suggest that OA-related disuse may act in synergism with aging-related muscle wasting by progressively redistributing the myofiber composition favoring T2 atrophied fibers as ROM worsens. Moreover, the data helps establish a platform for future studies on the similarities between OA AGs and OA QF to determine the AG as a surrogate for the QF. For further studies on OA AGs, we must address several limitations of the study related to sample collection, controls, and analysis. The AG is a small muscle with various insertion sites distal into the suprapatellar bursa and capsule, and proximal into the vastus intermedius with some components into the vastus medialis. As such, this complex interface leads to the imaging of the AG fibers at various angles. Therefore, fiber orientation when processing for analysis of CSA must be highly scrutinized. Also, the transition from T1 to T2 myofibers in the QF has been reported to accompany an increase in T2a/x hybrids^7,13^. These hybrids are linked to sedentary behavior and disuse-mediated atrophy^20^, and are considered one of the hallmarks for poor muscle health^8,14^. Even though our cross-sectional study suggests that decreasing T1 with correspondingly increasing T2 fibers are associated with worsening ROM, and higher FxC, further analysis warrants co-detection of T1 with T2a and T2x subtypes to assess poor muscle health from a histological perspective. Finally, subsampling of the AG during TKA should be refined to exclude closely associated, but contaminating tissue components (e.g., synovium and adipose tissue) to ensure pure muscle sample fixation for immunohistochemical screening, and future cryopreservation for complementary targeted analysis of transcripts associated with aberrant myofiber type switching, atrophy, and fibrosis.

## Methods

### Patients and ROM

This study was approved by the Louisiana State University Health-New Orleans Institutional Review Board (IRB no. 9630) and all methods were performed in accordance with relevant guidelines and regulations. Informed consent was obtained from all patients prior to participation. Participants were adults (≥ 18 years) diagnosed with clinical and radiographic signs and symptoms of knee OA and therefore eligible for TKA. TKA was performed on all patients in the study sample by the same fellowship-trained arthroplasty surgeon between October 2017 and March 2020 using standard surgical technique and equivalent implants. All patients received the same rapid recovery peri-operative protocols.

Variables obtained from patients’ medical charts include race, age, sex, body mass index (BMI), KL radiographic grade, and ROM. ROM was measured preoperatively with a goniometer for active flexion (145° maximum) and extension, and both reported in 5°intervals. Patient-reported outcome entries from the symptoms, pain, activities of daily life (ADL), and quality of life (QOL) subscales of the KOOS questionnaire were collected within two weeks prior to surgery. KOOS scores ranging from 0 (worse) to 100 (best) were calculated and stored in an encrypted database. The patient sample was divided into three groups of empirical ranges of total ROM: (1) ≤ 85° (poor); (2) 90°–110° (fair); and (3) ≥ 115° (good), considering 110° as the minimum extent of flexion required for most activities of daily life^21,22^. The patient sample was also grouped by (1) low (< 5°), (2) moderate (5°–10°), and (3) high (> 10°) FxC measurements considering that individuals with ≥ 10° FxC are likely to manifest clinically significant extension deficits^23^.

### AG collection, histology, and immunohistochemistry

AGs were isolated from the discarded tissue when performing routine anterior compartment synovectomy during total knee arthroplasty, fixed in zinc-buffered formalin for 4 days, paraffin processed, embedded with longitudinal fibers perpendicular to the cutting plane of the microtome, and sectioned at 4 µm onto positively charged slides. Sections were dried at 60 °C for 45 min, deparaffinized, submerged in pH 6.0 citrate buffer (Abcam, Cambridge, UK) at 60 °C for 17 h, and allowed to cool at room temperature for 20 min. Slides were washed in phosphate-buffered saline where indicated. Following treatment with Protein Block (Abcam) for 15 min, sections were co-incubated overnight at 4 °C with antibodies against slow MHC (Abcam; mouse monoclonal; 5 µg/mL) and fast MHC (Abcam; rabbit polyclonal; 2 µg/mL) to co-detect all isoforms of T1 and T2 myofibers^13^, respectively, and washed. Slides were co-incubated for 45 min at room temperature with anti-mouse and anti-rabbit F(ab’)2 secondary antibodies (Jackson Immunoresearch, West Grove, PA, US; goat polyclonal; 4 µg/mL) conjugated to Alexa 488 and Alexa 594, respectively, and washed. Slides were cover-slipped with Prolong Diamond (Thermo Fisher, Waltham, MA, US).

### Confocal imaging and myofiber measurements

We captured two fields (50–125 fibers each) per sample with an FV1000 laser scanning confocal microscope (Olympus of America, Center Valley, PA, US) at 200 × magnification using 405, multi-Argon, and 592 laser lines with respective photodetector units in sequential mode to detect autofluorescence, slow MHC + Alexa 488, and fast MHC + Alexa 594 labels, respectively. The perimeters of labeled T1 and T2 myofibers were segmented and individualized with a watershed separation algorithm, automatically counted, and individual myofiber CSA measured in µm^2^, using Slidebook 5.0 software (Intelligent Imaging innovations, Denver, CO, US), with the autofluorescence from the unstained endomysium serving as the interface between labeled fibers.

### Statistical analyses

GraphPad PRISM 9 (GraphPad Software, La Jolla, CA) was used for all analyses. Comparison between means of BMI, KL scores, KOOS, fiber distribution, and fiber CSAs for participants grouped by ROM were tested using Student t-test, Kruskal–Wallis, or one-way ANOVA with Tukey’s post hoc test. The association between T2 fiber percentages, CSA, or KOOS and ROM values for the entire patient sample was determined by Spearman’s correlation coefficient ρ. Alpha was set to 0.05 for all analyses.
